# Salt-Stress Response Mechanisms Using de Novo Transcriptome Sequencing of Salt-Tolerant and Sensitive *Corchorus* spp. Genotypes

**DOI:** 10.3390/genes8090226

**Published:** 2017-09-18

**Authors:** Zemao Yang, Ruike Lu, Zhigang Dai, An Yan, Qing Tang, Chaohua Cheng, Ying Xu, Wenting Yang, Jianguang Su

**Affiliations:** 1 Institute of Bast Fiber Crops, Chinese Academy of Agricultural Sciences/Key Laboratory of Stem-fiber Biomass and Engineering Microbiology, Ministry of Agriculture, Changsha 410125, China; ysyzm@aliyun.com (Z.Y.); luruike33@163.com (R.L.); dzgmonkey@126.com (Z.D.); qingtang1996@163.com (Q.T.); chengchaohua@sohu.com (C.C.); xuying0420@163.com (Y.X.); 2 Natural Sciences and Science Education, National Institute of Education, Nanyang Technological University, Singapore 637616, Singapore; an.yan@nie.edu.sg; 3 Key Laboratory of Crop Physiology, Ecology and Genetic Breeding, Ministry of Education, Jiangxi Agricultural University, Nanchang 330045, China; wtyang@jxau.edu.cn

**Keywords:** ABA signaling, *Corchorus*, differentially expressed unigenes, methionine metabolism, salt stress, transcriptome

## Abstract

High salinity is a major environmental stressor for crops. To understand the regulatory mechanisms underlying salt tolerance, we conducted a comparative transcriptome analysis between salt-tolerant and salt-sensitive jute (*Corchorus* spp.) genotypes in leaf and root tissues under salt stress and control conditions. In total, 68,961 unigenes were identified. Additionally, 11,100 unigenes (including 385 transcription factors (TFs)) exhibited significant differential expression in salt-tolerant or salt-sensitive genotypes. Numerous common and unique differentially expressed unigenes (DEGs) between the two genotypes were discovered. Fewer DEGs were observed in salt-tolerant jute genotypes whether in root or leaf tissues. These DEGs were involved in various pathways, such as ABA signaling, amino acid metabolism, etc. Among the enriched pathways, plant hormone signal transduction (ko04075) and cysteine/methionine metabolism (ko00270) were the most notable. Eight common DEGs across both tissues and genotypes with similar expression profiles were part of the PYL-ABA-PP2C (pyrabactin resistant-like/regulatory components of ABA receptors-abscisic acid-protein phosphatase 2C). The methionine metabolism pathway was only enriched in salt-tolerant jute root tissue. Twenty-three DEGs were involved in methionine metabolism. Overall, numerous common and unique salt-stress response DEGs and pathways between salt-tolerant and salt-sensitive jute have been discovered, which will provide valuable information regarding salt-stress response mechanisms and help improve salt-resistance molecular breeding in jute.

## 1. Introduction

Salt stress is a major environmental stressor that restricts plant development and growth, resulting in considerable crop yield losses worldwide [1,2]. Environmental pollution and climate change has intensified the adverse effects of salt stress through raising soil salinity. Thus, continued agricultural productivity and environmental conservation urgently depend on improving plant tolerance to salt through identifying mechanisms underlying salt-stress responses.

Salt stress exerts both primary and secondary effects on the plant. The former involves shifts in osmotic dynamics or ion content, leading to ion toxicity, whereas the latter is more complex and includes oxidative stress, damage to cellular components (e.g., membrane nucleic acids or lipids), and metabolic dysfunction [1]. These effects trigger a cascade of signal transduction pathways that alter gene expression profiles, membrane trafficking, energy metabolism, protein phosphorylation/dephosphorylation, and hormonal levels [3].

The synthesis of these salt-stress-induced compounds in plants occurs through a complex network of multiple pathways. Notably, the phytohormone abscisic acid (ABA) is involved in a signaling pathway central to salt stress responses [1]. First, the plasma membrane NADPH oxidase RbohF generates H_2_O_2_ to modulate calcium signals that affect the downstream ABA response [4]. Abscisic acid binds to PYL receptors and PP2C proteins, forming a PYL-ABA-PP2C complex that activates SnRK2s [5]. The latter group of protein kinases phosphorylates bZIP and other transcription factors (TF) [6] that regulate antioxidant production. For example, overexpressing the TF IbMYB1 leads to anthocyanin accumulation and enhances salt stress tolerance in the transgenic potato (Solanum tuberosum) [7]. Additionally, ABA can activate mitogen-activated protein kinase (MAPK) pathways to phosphorylate many other ABA effector proteins [4].

Although reactive oxygen species (ROS) function in plant signal-transduction pathways [8], osmotic stress (including high salinity) can cause excessive ROS accumulation, leading to oxidative damage. Thus, plants have evolved mechanisms to respond to ROS damage. For instance, expansins and xyloglucan-modifying enzymes are expressed to repair cell walls that have stiffened from salinity-induced oxidative stress [9]. As another example, the ROS-mediated oxidation of methionine to methionine sulfoxide (MetO) alters protein conformation and activity, which then ameliorates oxidative damage; meanwhile, the Met biosynthetic pathway also involves the intermediate S-adenosylmethionine (SAM) as a primary methyl donor, which further yields S-adenosyl-l-homocysteine that is metabolised to eventually regenerate Met, which may reinforce the pivotal role of Met in the plant stress response [10]. Furthermore, plants produce carotenoids and phenolics (i.e., phenolic acids and flavonoids), secondary metabolites that act as ROS-scavenging antioxidants [11] to attenuate oxidative damage.

Jute (*Corchorus*) refers to two cultivated species, *Corchorus capsularis* L. and *Corchorus olitorius* L., which are among the most important bast fiber crops in the world [2]. Global demand for jute has recently increased because of its broad-spectrum application and eco-friendly characteristics [12,13]. Improving jute salt tolerance will expand its cultivation in saline environments and reduce competition with food crops for arable land. Although several studies are available on the morphological, physiological, and proteomic components of salt tolerance in jute [2], molecular research, particularly transcriptome studies, is limited [14]. The data are critical for a full understanding of salt-stress response mechanisms in jute, necessary for targeted genetic modification or breeding efforts to improve salt tolerance. Therefore, in the present study, we conducted high-throughput sequencing on 24 transcriptomes in leaves and roots from salt-sensitive and salt-tolerant jute genotypes. The objective was to systematically identify potential salt-stress responsive genes. The results will clarify regulatory pathways involved in jute salt tolerance.

## 2. Materials and Methods

### 2.1. Plant Materials and Salt-Stress Treatment

Two *C. olitorius* L. genotypes, NY (salt-stress sensitive) and TC (salt-stress tolerant), were hydroponically cultivated (in Yoshida nutrient solution, Yuchuan Microbial Products, Tianjing, China) at 25–28 °C in a greenhouse. Each genotype was grown in two containers containing 30 plants per container. At the nine-leaf stage, three uniform seedlings were selected from every container and transferred to four new containers (one control and one salt-stress treatment for each genotype). Control containers contained only Yoshida nutrient solution, while salt-stress containers contained the solution supplemented with 250 mM NaCl. After 12 h, the roots and leaves from every plant were collected for RNA extraction.

### 2.2. RNA Isolation

Total RNA was isolated from tissue samples using Trizol (Invitrogen, Santa Clara, CA, USA), following manufacturer protocol. RNA degradation and contamination was monitored in 1% agarose gels. RNA purity was confirmed using the NanoPhotometer^®^ spectrophotometer (Implen, West Lake Village, CA, USA), and the concentration was measured using the Qubit^®^ RNA Assay Kit in Qubit^®^ 2.0 Flurometer (Life Technologies, Carlsbad, CA, USA). RNA integrity was assessed with the RNA Nano 6000 Assay Kit of the Agilent Bioanalyzer 2100 system (Agilent Technologies, Palo Alto, CA, USA). Twenty-four samples were used for transcriptome sequencing (NYCR, NY control root; NYCL, NY control leaf; NYSR, NY salt-stressed root; NYSL, NY salt-stressed leaf; TCCR, TC control root; TCCL, TC control leaf; TCSR, TC salt-stressed root; TCSL, TC salt-stressed leaf). For each sample, three independent biological replicates were performed.

### 2.3. Transcriptome Sequencing

Twenty-four RNA sequencing libraries were generated using NEBNext^®^ Ultra™ RNA Library Prep Kit for Illumina^®^ (New England Biolabs, Ipswich, MA, USA), following manufacturer protocol. Index codes were added to link sequences with each sample. Library quality was assessed on the Agilent Bioanalyzer 2100 system. Index-coded samples were clustered on a cBot Cluster Generation System using TruSeq PE Cluster Kits v3-cBot-HS (Illumina, San Diego, CA, USA) for RNA libraries. After cluster generation, library preparations were sequenced on an Illumina HiSeqTM 4000 platform, and paired-end reads were generated for transcriptome sequencing. All sequencing data were deposited into the NCBI SRA database under accession number SRP116874.

### 2.4. Transcriptome Data Analysis and Annotation

Low-quality reads and those containing adapter or ploy-N were removed from raw data using in-house Perl scripts. All subsequent analyses were performed on high-quality clean data. Transcriptome assembly for cleaned data was performed in Trinity [15] with default parameters (except min_kmer_cov set to 2). In addition, after obtaining the transcripts, all clean reads were mapped to the transcripts and the transcripts with less than 5× coverage were removed. Gene function was annotated using the following databases: nr (non-redundant database; NCBI) [16], nt (nucleotide collection database; NCBI,) [17], KO (Kegg Orthology) [18], GO (Gene Ontology) [19], KOG (Eukaryotic Orthologous Groups), [20] Pfam [21], and SwissProt [22] using BLASTx with an E-value threshold of 10^−5^ [23].

### 2.5. Identification and Biological Analysis of Differentially Expressed Genes

Gene expression levels for each sample were estimated using RSEM [24]. Clean data were mapped back onto the assembled transcriptome and a read count per gene was obtained from the mapping results. Differential expression analysis of the treatment and control groups was performed using the DESeq R package (1.10.1) [25], with an adjusted *p* < 0.05. Gene ontology (GO) analysis of the DEGs was performed with GOseq R packages based on the Wallenius noncentral hypergeometric distribution [26]. KOBAS software [27] was used to test the DEG statistical enrichment of KEGG pathways.

### 2.6. Real-Time Quantitative PCR Analysis

To validate RNA-seq results, the gene expression from nine randomly selected DEGs was analyzed using a two-step real-time quantitative PCR (qRT-PCR). Two independent biological and three technical replicates were performed. First, 1 μg total RNA per sample was reverse-transcribed into first-strand cDNA using the M-MuLV Reverse Transcriptase kit (Fermentas, Burlington, ON, Canada), following manufacturer protocol. After 10× dilution, cDNA was used as templates for qRT-PCR (AB GeneAmp PCR System 9700;Applied Biosystems, Foster City, CA, USA). The reaction mixture was prepared using the FastStart Universal SYBR Green Master (ROX) kit (ROCHE) following manufacturer protocol and added to an optical 384-well plate. The jute ELF gene was selected as the endogenous control [28]. Primers for the DEGs and ELF are listed in Supplementary File S1. Relative expression levels were determined using the comparative Ct method [29].

## 3. Results

### 3.1. Transcriptome Sequencing and Assembly

Transcriptome sequencing and analysis of 24 NY (salt-sensitive genotype) and TC (salt tolerant) samples generated 1,245,748,588 raw reads (Table 1). We obtained 1,204,397,278 (96.68%) clean reads with an average GC content of 44.61% and high base quality scores (Q20 > 97%), resulting in 180.68 Gb of sequencing data for transcriptome de novo assembly. Clean reads were used to assemble 134,793 transcripts and 68,961 non-redundant unigenes. Transcripts were used as reference sequences for downstream analysis, and over 80% of the clean reads for each sample were mapped into reference transcripts. Transcript and unigene lengths varied between 201 and 15,890 nucleotides, with average lengths of 1322 and 1600, respectively (Table 2).

### 3.2. Unigene Functional Annotation

**Figure 1:**
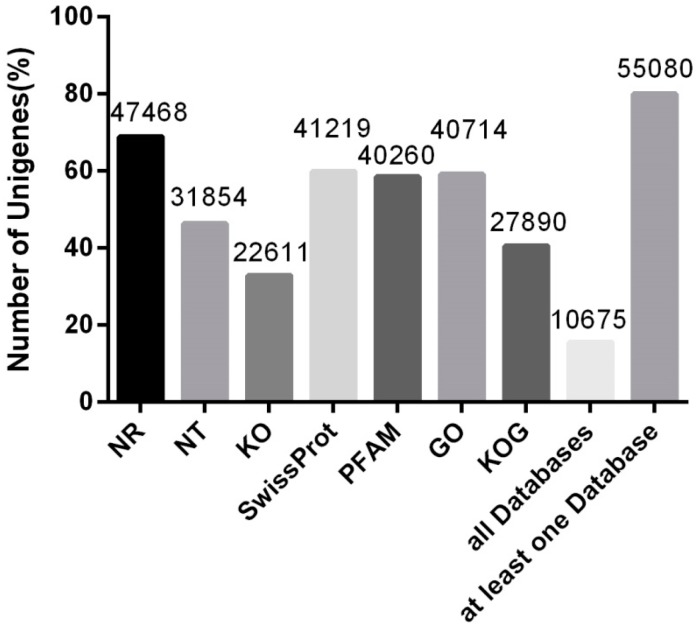
Unigene annotation success rates across multiple databases.

Of the 68,961 unigenes, 10,675 (15.47%) were annotated in all seven databases (NR, NT, KO, GO, KOG, Pfam, and SwissProt); 55,080 (79.87%) unigenes were successfully annotated in at least one database. Most unigenes (47,468; 68.83%) were aligned with the NR database, followed by SwissProt (41,219; 59.77%) and GO (40,714; 59.03%). Only 22,611 (32.78%) unigenes were matched with KO (Figure 1). Respectively, GO, KOG, and KO analyses assigned matched unigenes to 56 (Supplementary Figure S1), 26 (Supplementary Figure S2), and 19 (Supplementary Figure S3) groups.

### 3.3. Differential Gene Expression in Response to Salt-Stress Treatments

**Figure 2:**
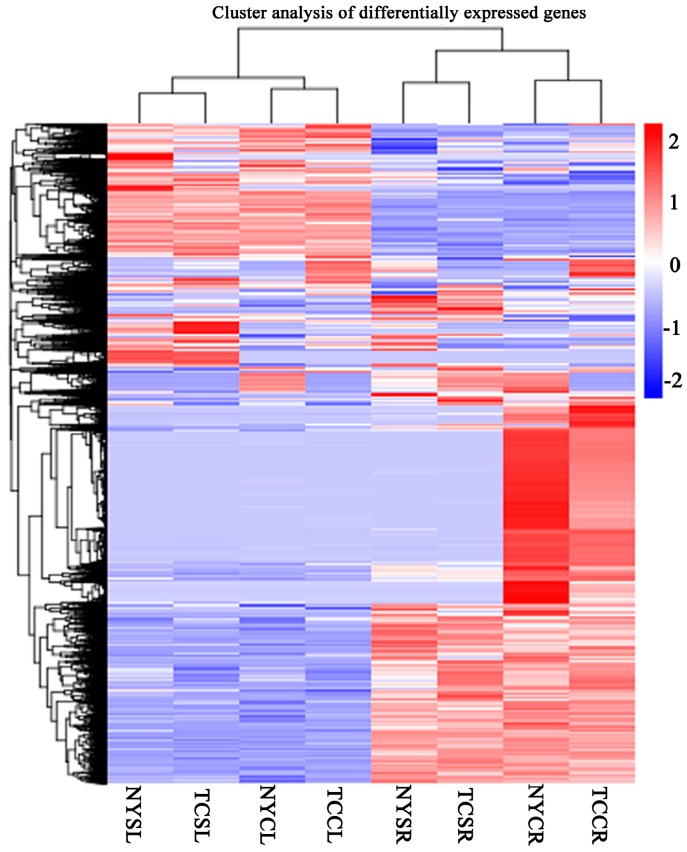
Cluster analysis of differential gene expression profiles in NYCR, NY control root; NYCL, NY control leaf; NYSR, NY salt-stressed root; NYSL, NY salt-stressed leaf; TCCR, TC control root; TCCL, TC control leaf; TCSR, TC salt-stressed root; and TCSL, TC salt-stressed leaf.

**Figure 3:**
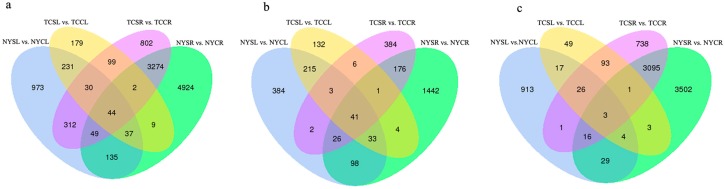
Statistics of differentially expressed genes (DEGs). (**a**) all of DEGs; (**b**) upregulated DEGs; and (**c**) downregulated DEGs in various NY and TC comparison groups under salt stress and control conditions.

Differential gene expression analysis of tissues in NY and TC under salinity stress and control conditions revealed 11,100 differentially expressed unigenes (DEGs) (Supplementary File S2). Overall, differential gene expression profiles were more similar within the same tissue type, rather than between genotypes (Figure 2). In NY, 1811 and 8427 significant DEGs were present in leaf and root tissues, respectively. Fewer DEGs were observed in TC tissues (631 and 4612 in leaves and roots, respectively) (Figure 3). Next, comparative transcriptome analysis found that 265 and 175 common DEGs across the two tissues (leaf and root) were identified in NY and TC, respectively. Similarly, 342 and 3369 DEGs (in leaves and roots, respectively) were common across both NY and TC (Figure 3). In addition, 44 DEGs were common across both tissues and genotypes (Supplementary File S3, Figure 3). Ninety-eight DEGs were only present in TC leaves and roots (Supplementary File S4, Figure 3).

### 3.4. Differential Expression of Transcription Factors

We identified 2372 transcription factors (TFs) from 79 families (Supplementary File S5), with 385 differentially expressed TFs (56 families) under salt stress (Supplementary File S6). Over 58% of the identified TFs belonged to the MYB, Orphans, CCAAT, AP2-EREBP, FHA, C3H, bHLH, C2H2, WRKY, HSF, NAC, and bZIP TF families, with the most common being MYB (45 differentially expressed TFs). Most were already implicated in various abiotic stress-response pathways of different plants. Under salinity stress, 54 (30/24), 323 (124/199), 18 (14/4), and 109 (25/84) upregulated/downregulated TFs were present in NY leaves (NYL), NY roots (NYR), TC leaves (TCL), and TC roots (TCR), respectively.

### 3.5. Functional Categorization of DEGs in Salt-Stressed Jute

Gene ontology analysis showed that DEG enrichment categories differed by tissue (leaf versus root) and genotype (NY versus TC) (Supplementary Figure S4). Numerous terms were involved in the salt stress response. For example, amide biosynthetic processes were mostly enriched in NYL and TCR. In addition, the terms structural molecular activity, intracellular non-membrane-bounded organelle etc. were significantly enriched in three versus groups (NYSL_vs._NYCL, TCSL_vs._TCCL and TCSR_vs._TCCR). Transmembrane receptor protein tyrosine kinase activity and calcium ion binding were also highly enriched, especially in the roots.Xyloglucan translation and metabolism enrichment was specific to NYL. Enrichment of protein phosphorylation, protein kinase activity, and ATP binding was specific to NYR. Proteolysis, dynein activator complex, cysteine-type peptidase activity, and hydrolase activity were significantly enriched in TCR. Finally, riboflavin metabolic processes and acid phosphatase activity were significantly enriched in TCL.

### 3.6. Metabolic Pathways Enhanced under Salt Stress Conditions

**Figure 4:**
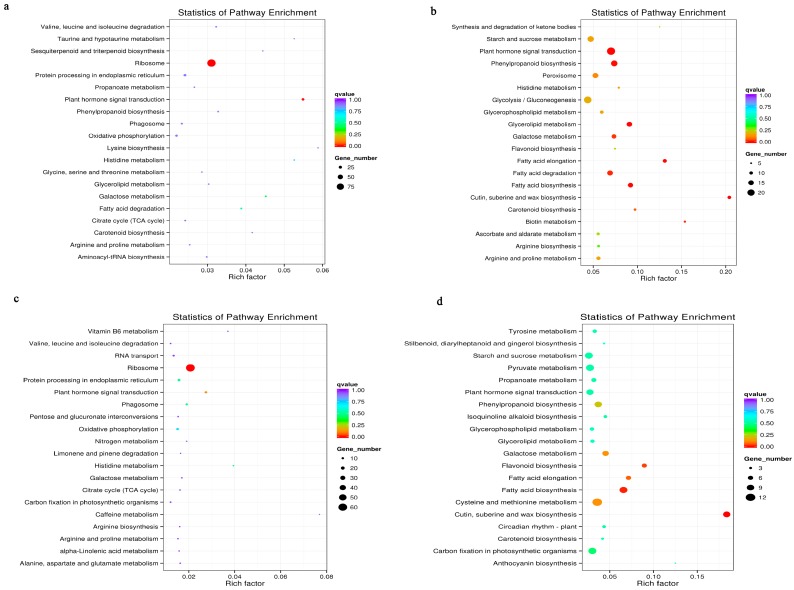

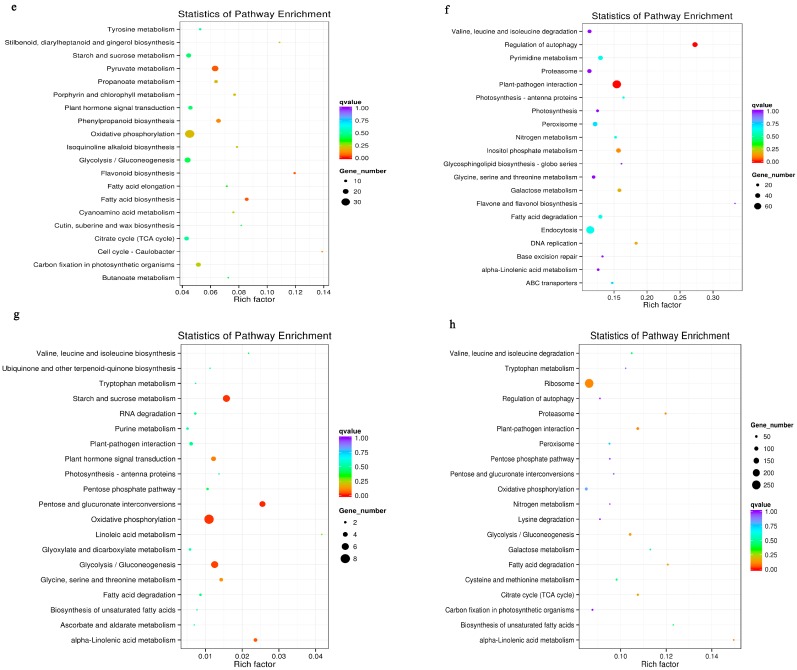
Metabolic pathways enrichment under salt stress conditions. Enriched pathways of upregulated unigenes (**a**–**d**); (**a**) Enriched pathways of upregulated unigenes in NY leaves (NYL) underlying salt treatment vs. control; (**b**) Enriched pathways of upregulated unigenes in NY roots (NYR) underlying salt treatment vs. control; (**c**) Enriched pathways of upregulated unigenes in TC leaves (TCL) underlying salt treatment vs. control; (**d**) Enriched pathways of upregulated unigenes in TC roots (TCR) underlying salt treatment vs. control. Enriched pathways of downregulated unigenes (**e**–**h**); (**e**) Enriched pathways of downregulated unigenes in NY leaves (NYL) underlying salt treatment vs. control; (**f**) Enriched pathways of downregulated unigenes in NY roots (NYR) underlying salt treatment vs. control; (**g**) Enriched pathways of downregulated unigenes in TC leaves (TCL) underlying salt treatment vs. control; (**h**) Enriched pathways of downregulated unigenes in TC roots (TCR) underlying salt treatment vs. control.

We compared the first 20 most enriched pathways among the NYL, NYR, TCL, and TCR treatment groups (Figure 4) to identify those that were well represented under salt stress. Plant hormone signal transduction was enriched in all four treatment groups. Cutin, suberin, and wax biosynthesisstarch and sucrose metabolism pathways were significantly enriched by 28 upregulated unigenes in the roots (of both genotypes), and by 23 downregulated unigenes in the leaves. Sesquiterpenoid and triterpenoid biosynthesis was only enriched in NYL, while inositol phosphate metabolism was enriched in NYR. Furthermore, 20, 70, and 30 downregulated unigenes for DNA replication, endocytosis, and photosynthesis, respectively, as well as eight upregulated unigenes related to ascorbate and aldarate metabolism, were significantly enriched only in NYR. Seven downregulated and four upregulated unigenes involved in the isoquinoline alkaloid biosynthesis pathway were enriched in NYL and TCR, respectively. Finally, numerous DEGs in TC (regardless of tissue) significantly enriched pathways linked to cysteine and methionine metabolism (45 DEGs), pentose metabolism (47 DEGs), and unsaturated fatty-acid biosynthesis and tryptophan metabolism (17 downregulated genes).

**Figure 5:**
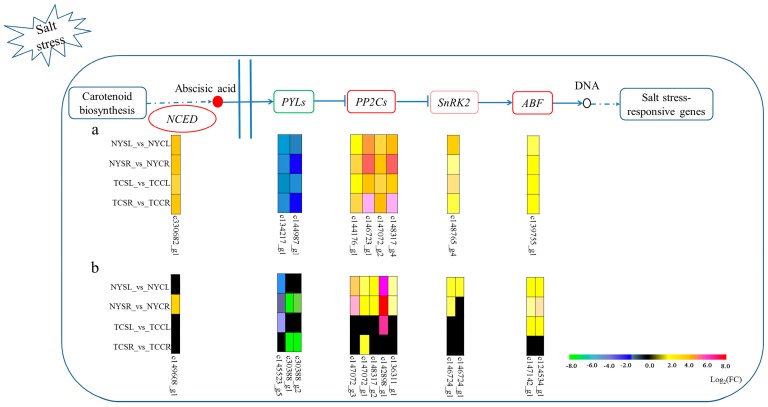
PYL-ABA-PP2C pathway in plant hormone signal transduction. (**a**) Expression profile of nine common DEGs across both tissues and genotypes; (**b**) Expression profile of twelve DEGs in NY leaves, NY roots, TC leaves, or TC roots. NYSL (NY salt-stressed leaf); NYCL (NY control leaf); NYSR (NY salt-stressed root); NYCR (NY control root); TCSL (TC salt-stressed leaf); TCCL (TC control leaf); TCSR (TC salt-stressed root); TCCR (TC control root). NCED: Nine-cis-epoxycarotenoid dioxygenase; SnRK2: SNF1-related protein kinase 2; ABF: ABA responsive element binding factor; ABA: abscisic acid; PYL: pyrabactin resistant-like/regulatory components of ABA receptors; PP2C: protein phosphatase 2C.

Among the enriched pathways, plant hormone signal transduction (ko04075) and cysteine/methionine metabolism (ko00270) were the most notable. Of the 44 common DEGs across both tissues and genotypes, 12 had an unknown function and the remainder were generally related to abiotic stress responses. Of them, eight were part of the PYL-ABA-PP2C pathway: four PP2C (upregulated, 3.78~126.23 fold change), two PYL (downregulated, −3.40~−30.350 fold change), one SnRK2 (SNF1-related protein kinase 2; upregulated, 2.10~12.24 fold change), and one ABF (ABA responsive element binding factor; upregulated, 3.00~5.01). Additionally, one NCED3 (nine-cis-epoxycarotenoid dioxygenase; upregulated, 11.72~25.11 fold change) needed for ABA synthesis was also included in the 44 DEGs (Figure 5a). Another 13 DEGs which were only included in a part of four treatment groups had similar expression profiles (three PYL/PYR, all downregulated, −1.73~−250.28 fold change; five PP2Cs, all upregulated, 2.21~152.93 fold change; two SnRK2, both upregulated, 2.54~3.17 fold change; two ABF, both upregulated, 2.35~5.15 fold change; one NCED1 gene upregulated, 9.35 fold change) (Figure 5b).

**Figure 6:**
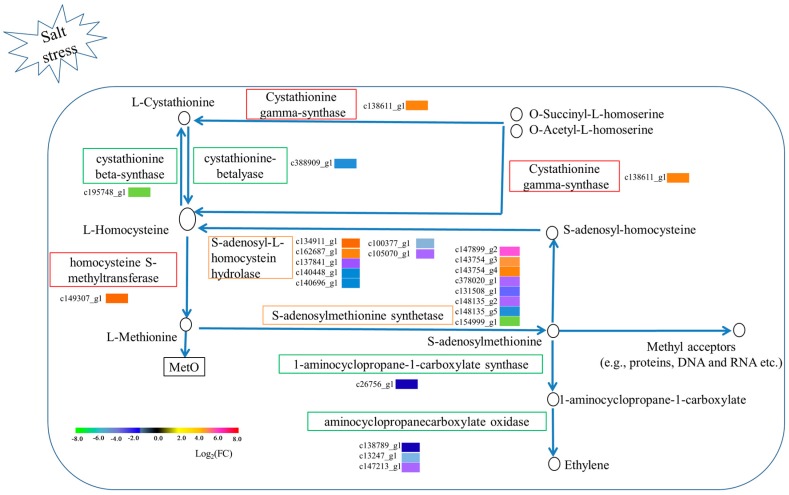
Methionine metabolism pathway in TC root tissue and the expression profile of 23 DEGs in the pathway.

The cysteine and methionine metabolism pathway was only enriched in TC root tissue. Twenty-three DEGs were involved in methionine metabolism: one cystathionine gamma-synthase (upregulated, 18.81 fold change), one cystathionine beta-lyase (downregulated, −40.36 fold change), one beta-synthase (downregulated, −92.32 fold change), one homocysteine S-methyltransferase (upregulated, 26.91 fold change), eight S-adenosylmethionine synthetase (three upregulated, 13.67~64.67 fold change; five dowregulated, −11.28~−150.33), seven S-adenosyl-l-homocysteine hydrolase (two upregulated, 15.26~29.36 fold change; five downregulated, −14.35~−60.01 fold change), one 1-aminocyclopropane-1-carboxylate synthase (part of ethylene biosynthesis; downregulated, −2.83 fold change), and three aminocyclopropanecarboxylate oxidase (part of ethylene biosynthesis; downregulated, −2.94~−73.84 fold change; DEG c147213_g1 was expressed only in TC) (Figure 6).

### 3.7. Validation of DEGs

**Figure 7:**
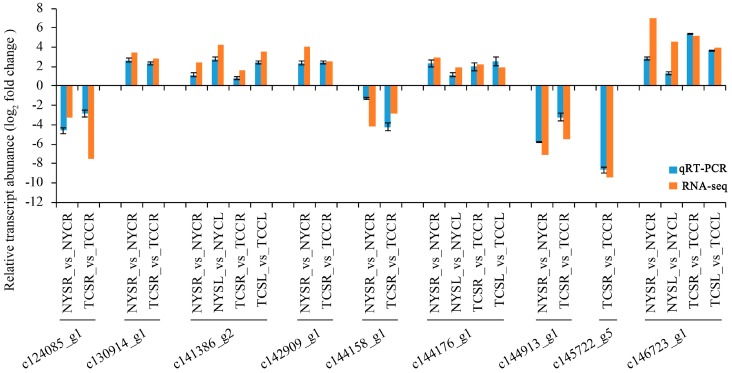
Quantitative real-time PCR (qRT-PCR) validation analysis of RNA sequencing of nine DEGs. Data represent log2(fold changes) to each DEG’s relative transcript abundance in NYSR (NY salt-stressed root) vs. NYCR (NY control root); NYSL (NY salt-stressed leaf) vs. NYCL (NY control leaf); TCSR (TC salt-stressed root) vs. TCCR (TC control root); TCSL (TC salt-stressed leaf) vs. TCCL (TC control leaf). The blue bars represent the expression via qRT-PCR and red bars represent expression via RNA-seq.

We performed qRT-PCR analysis to validate the results of differential gene expression obtained from the RNA-seq data. A total of nine unigenes were selected randomly for qRT-PCR analysis. The qRT-PCR was performed in the roots and leaves of NY and TC under treatment and control. The qRT-PCR analysis validated the results generated from high-throughput sequencing (Figure 7). The PCR- and RNA-seq-generated expression profiles of the DEGs were very similar (correlation coefficient of 0.89, Supplementary File S7).

## 4. Discussion

Differential gene expression analysis revealed that salt-tolerant jute had fewer DEGs than salt-sensitive jute did, similar to results from drought stress studies in other plants [30,31]. Furthermore, the roots of both genotypes had more DEGs than leaves, suggesting that roots are the primary organ involved in the salt-stress response. The PYL-ABA-PP2C pathway appeared to be particularly important, as we found eight DEGs in the salt-stressed tissues of both genotypes, corroborating several previous plant studies [8,32]. However, our results showed that PYL genes were downregulated, which somewhat contradicts the basic model of ABA signaling. Typically, PYLs bind to ABA and form PYL-ABA-PP2C complexes with PP2Cs, inhibiting the latter. This inhibition releases autophosphorylating SnRK2s, which then phosphorylate many downstream effectors [1]. Therefore, PYL expression is expected to keep pace with ABA concentration and be upregulated under stress. Several factors may explain PYL downregulation. First, increased PP2Cs:PYR/PYL levels may heighten SnRK2 activity, which inhibits PYLs [33]. Second, rapid increases of endogenous ABA might result in unkown interactions of ABA with PP2Cs, thus activating SnRK2 and inhibiting PYL expression. Third, ABA signaling could trigger downstream expression that negatively regulates ABA receptors such as PYLs. Further research is clearly necessary to explore these possible mechanisms. Further emphasizing the importance of ABA signaling to the salt-stress response, KEGG analysis revealed the enrichment of several related pathways (e.g., sesquiterpenoid, triterpenoid, and carotenoid biosynthesis). These results indicate that the ABA signaling pathway in response to salt stress is conserved across plants.

More differentially expressed TFs were present in NY than in TC plants. Previous studies have shown that the major TF families involved in abiotic stress responses are AP2/EREBP, MYB, WRKY, HSF, NAC, and bZIP [34,35]. Similarly, in this study, most TFs were from the MYB family, but we also found 23 Orphans family members. The Orphans have been reported in response to drought and PEG stresses in plants [36,37], but not in response to salt stress. Therefore, our results implied that Orphans TF families may also be important for salt stress tolerance in plants.

Numerous signal transduction pathways involving plant hormones and reactive oxygen species (ROS) have been implicated in the plant abiotic stress response [8,38]. In particular, ROS-response pathways are well represented (e.g., peroxisome, cysteine, and methionine metabolism) because salt stress exacerbates ROS production, overwhelming the antioxidant defense system and generating various secondary messengers under oxidative damage [1]. Interestingly, methionine metabolism was only enriched in TC root tissue. We found seven DEGs (up- and downregulated) encoding S-adenosylhomocysteine hydrolase (SAHH), which activates the methyl cycle through catalyzing the reversible hydrolysis of S-adenosylhomocysteine (SAH) to adenosine and homocysteine [3]. One DEG (upregulated) encoded homocysteine s-methyltransferase, which converts homocysteine to methionine. Under salt stress, the ROS-mediated oxidation of methionine to methionine sulfoxide (MetO) ameliorates oxidative damage [10]. Meanwhile, eight DEGs (three upregulated and five downregulated) encoded S-adenosylmethionine synthetases (SAMs), enzymes that convert methionine to the methyl donor, S-adenosylmethionine (SAM). Finally, SAM forms SAH via transferring methyl to acceptors (e.g., phospholipids, proteins, DNA, and RNA). Further transmethylation reactions are promoted via SAH-triggered feedback inhibition [3]. This process suggests that the seven up- and downregulated SAHHs and eight up- and downregulated SAMs may play different roles depending on methyl cycle cell internal environmental conditions, thus generating a feedback regulation of the methyl cycle.

Two enriched pathways appear to promote the methyl cycle through substrate synthesis and reducing substrate consumption (Figure 6). The first pathway converts O-Succinyl-l-homoserine and O-acetyl-l-homoserine directly into homocysteine (rather than through an initial conversion to l-cystathionine and then to homocysteine; notably, two unigenes encoding the l-cystathionine-to-homocysteine conversion were significantly downregulated) via the action of cystathionine gamma-synthase (encoded by an upregulated unigene).

Besides being involved in the methyl cycle, SAM can also be converted into ethylene by 1-aminocyclopropane-1-carboxylic acid (ACC) synthase and ACC oxidase (ACCO). However, one gene encoding ACC synthase and three encoding ACCO were downregulated in the study. This result implies that SAM was preferentially channeled towards the methyl cycle, and a similar result involved in biotic stress was revealed by a combination of proteomics and metabolomics in chickpea roots [39].

Both the methyl cycle and SAM may contribute to post-transcriptional changes in response to salt stress. Likewise, under salt stress, SAM methylates ROS acceptors (e.g., phospholipids, proteins, DNA, and RNA) to change their structure and activity. This process causes the epigenetic and post-transcriptional regulation of expression and translation, generating a response to salt stress. Overall, more DEGs and differentially expressed TFs were present in NY than in TC. These outcomes imply that epigenetic and post-transcriptional gene regulation may be primary contributors to the resistant response in salt-tolerant jute.

We also identified genes involved in cell wall biosynthesis, likely because excess ROS accumulation causes cell-wall stiffening. For example, cutin, suberin, and wax biosynthesis was enriched, in line with the role of wax as a major protective component that prevents excess water evaporation from the cell wall [34]. In addition, we observed the enrichment of metabolic pathways involving small biological molecules (e.g., arginine, proline) [40] that are crucial for plant adjustment to osmotic stress. Given the importance of these signaling pathways in the abiotic stress response, variation in salt-tolerance ability between NY and TC is likely due to the differential enrichment of specific pathways, notably those involved in the metabolism of cysteine, methionine, pentose, tryptophan, and unsaturated fatty acids. For instance, increased concentrations of unsaturated fatty acids in membrane lipids can enhance the tolerance of photosystem II to salt stress [41].

In summary, our study provides a comparative analysis of the transcriptome between salt-tolerant and salt-sensitive jute subjected to high salinity. Transcriptome analyses are still extremely rare in jute, despite the crop’s increasing agricultural importance and economic relevance. The results obtained in this analysis should contribute to an improved understanding of abiotic-stress tolerance in jute and facilitate inter-species comparisons for further clarity on plant tolerance mechanisms. Moreover, our data on DEGs should prove extremely useful for breeding programs to improve jute productivity under a variety of environments, including high salinity soils.

## Figures and Tables

**Table 1:** Raw and clean data from 24 NY (salt sensitive) and TC (salt tolerant) jute transcriptomes.

Genotypes	Tissues	Raw Reads (bp)		Clean Reads (bp)		Clean Bases (Gb)		Mapped Reads (%)		Q20	Avg. GC (%)
		Control	Salinity	Control	Salinity	Control	Salinity	Control	Salinity	(%)	
NY	Leaf	154,357,990	157,905,474	149,840,624	151,807,922	22.48	22.78	83.89%	83.84%	>97	44.61
	Root	170,983,540	150,144,930	165,732,832	145,221,480	24.86	21.78	80.72%	80.98%		
TC	Leaf	154,625,422	146,504,736	149,883,254	140,337,978	22.48	21.06	83.47%	83.22%		
	Root	147,290,466	163,936,030	142,892,496	158,680,692	21.44	23.8	81.06%	81.39%		
Total		1,245,748,588		1,204,397,278 (96.68%)		180.68		-	-		

**Table 2:** Assembly output statistics of all clean data in Trinity.

Parameters	Value
Number of unigenes	68,961
Number of transcripts	134,793
Average unigene length (bp)	1322
Average transcript length (bp)	1600
Unigene N50 (bp)	1721
transcript N50 (bp)	2263
Minimum unigene length (bp)	201
Minimum transcript length (bp)	201
Maximum unigene length (bp)	15,890
Maximum transcript length (bp)	15,890

## Supplementary Materials

The following are available online at www.mdpi.com/2073-4425/8/9/226/s1. Supplementary File S1. Primers for the DEGs and *ELF* gene, Supplementary File S2. All differentially expressed unigenes (DEGs) in NYSR (NY salt-stressed root) vs. NYCR (NY control root); NYSL (NY salt-stressed leaf) vs. NYCL (NY control leaf); TCSR (TC salt-stressed root) vs. TCCR (TC control root); TCSL (TC salt-stressed leaf) vs. TCCL (TC control leaf), Supplementary File S3. Differentially expressed unigenes (DEGs) were common across both tissues and genotypes, Supplementary File S4. Differentially expressed unigenes (DEGs) were present only in TC leaves and roots, not in NY, Supplementary File S5. Transcription factors (TFs) identified in the study, Supplementary File S6. Differentially expressed Transcription factors (TFs) identified in the study. Supplementary File S7. Result of correlation analysis between the RNA-seq and qRT-PCR analyses. Figure S1. Unigene classifications based on gene ontology analysis, Figure S2. Unigenes annotated by KOG, The horizontal axis is the name of clusters of KOG; and the vertical axis is the proportion of the group to the total number, Figure S3. Unigenes annotated by KO, The genes could be divided into five branches: cellular processes (A); Environmental Information Processing (B); genetic information processing (C); metabolic (D); and organismal system (E). Figure S4. Gene ontology analysis showed that GO terms enriched in NYSR (NY salt-stressed root) vs. NYCR (NY control root); NYSL (NY salt-stressed leaf) vs. NYCL (NY control leaf); TCSR (TC salt-stressed root) vs. TCCR (TC control root); TCSL (TC salt-stressed leaf) vs. TCCL (TC control leaf).
