# Supplementary figures and images for: Salt-Stress Response Mechanisms Using de Novo Transcriptome Sequencing of Salt-Tolerant and Sensitive Corchorus spp. Genotypes
Source: Genes (Basel). 2017 Sep 18;8(9):226. doi: 10.3390/genes8090226 (PMC5615359; doi:10.3390/genes8090226)

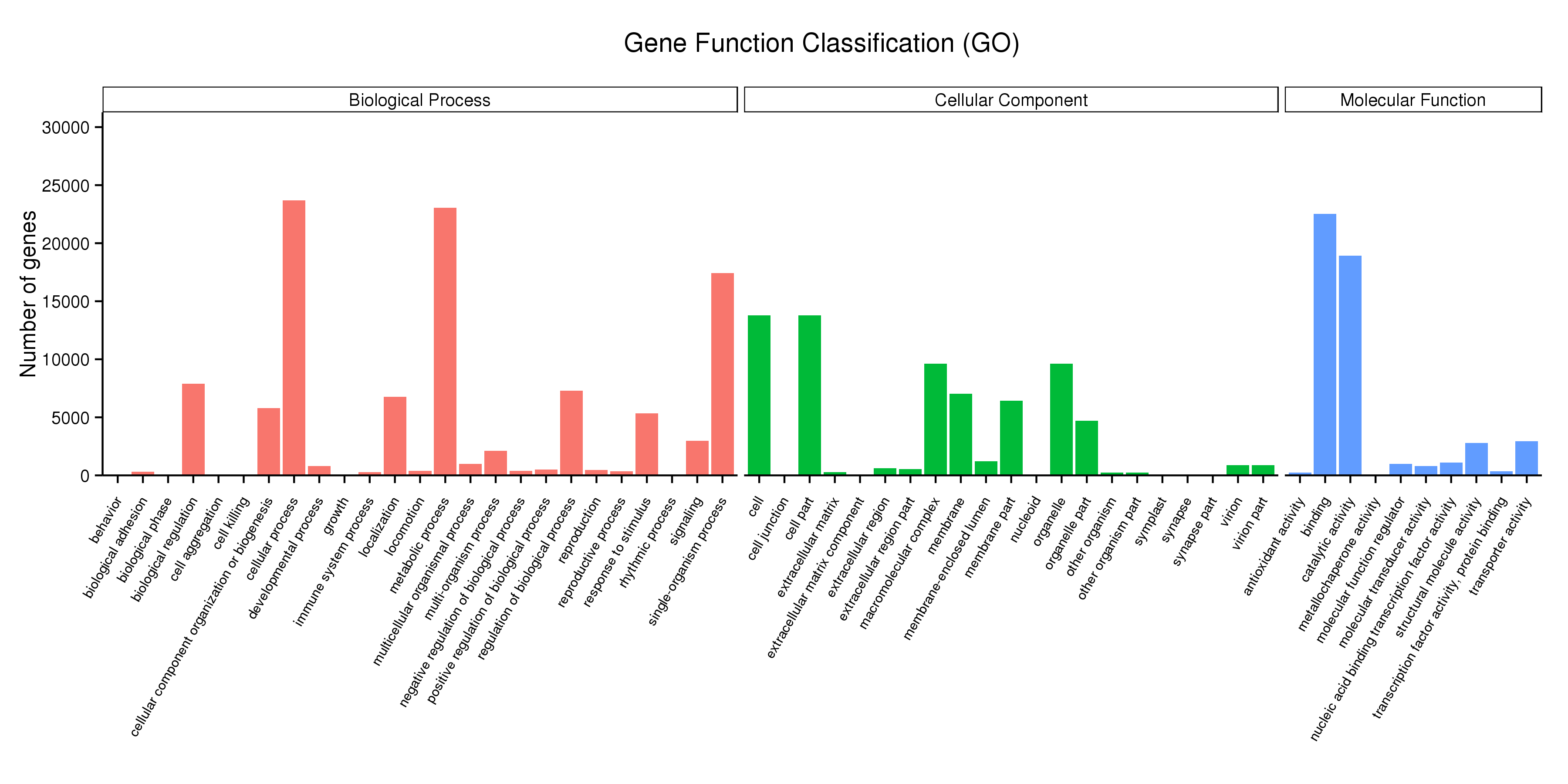

Supplement: Supplementary file 1 [file genes-08-00226-s001.zip › Supplementary Material/Supplementary figure S1.png]

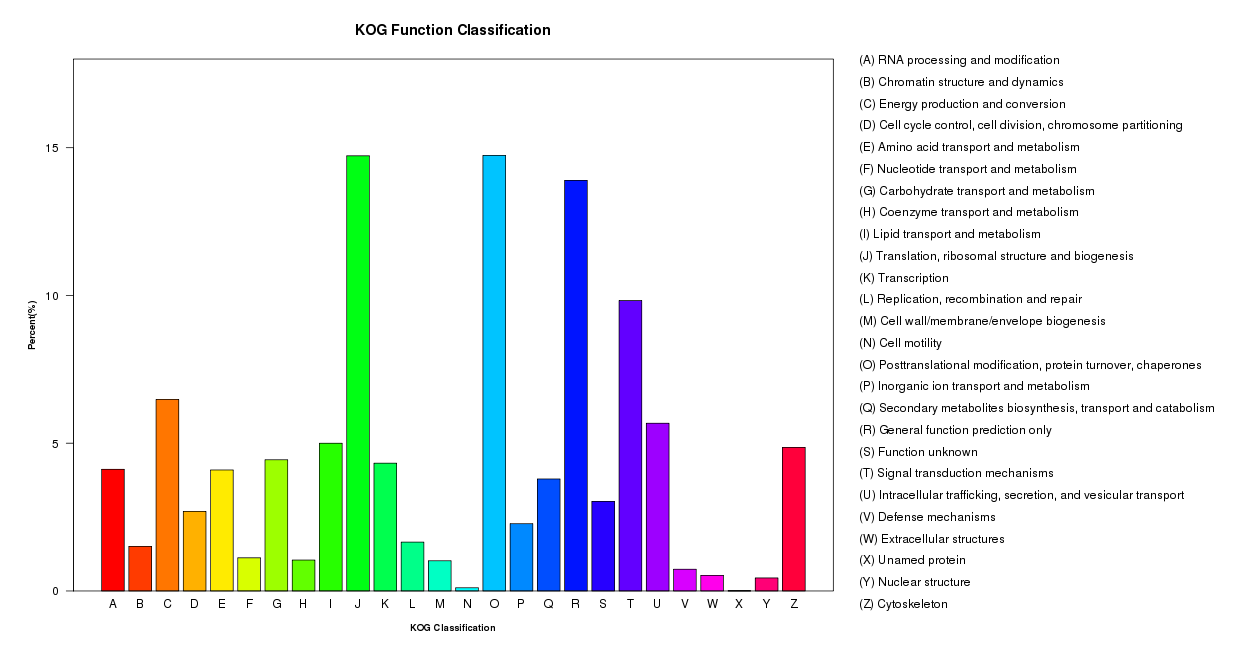

Supplement: Supplementary file 1 [file genes-08-00226-s001.zip › Supplementary Material/Supplementary figure S2.png]

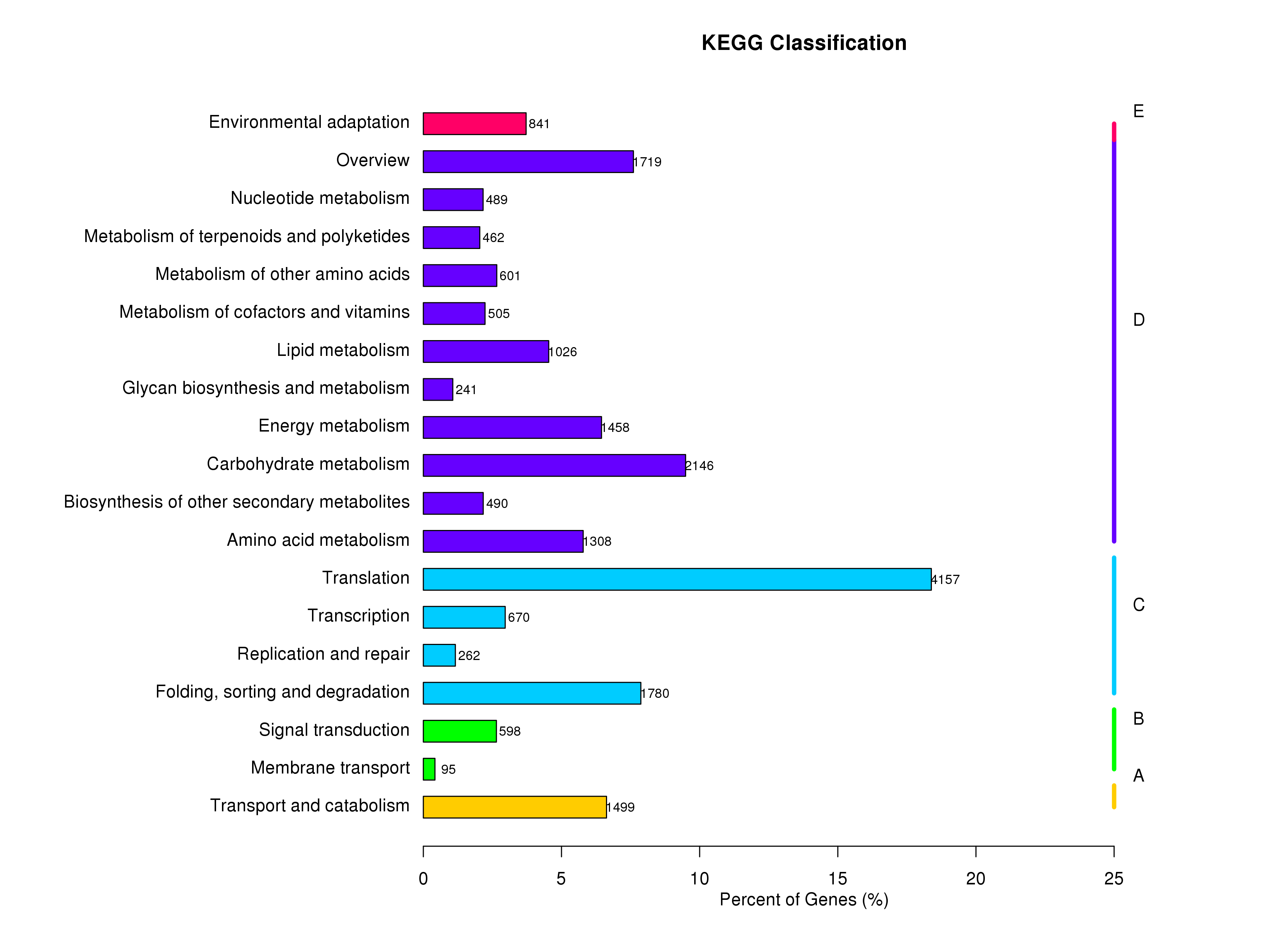

Supplement: Supplementary file 1 [file genes-08-00226-s001.zip › Supplementary Material/Supplementary figure S3.png]

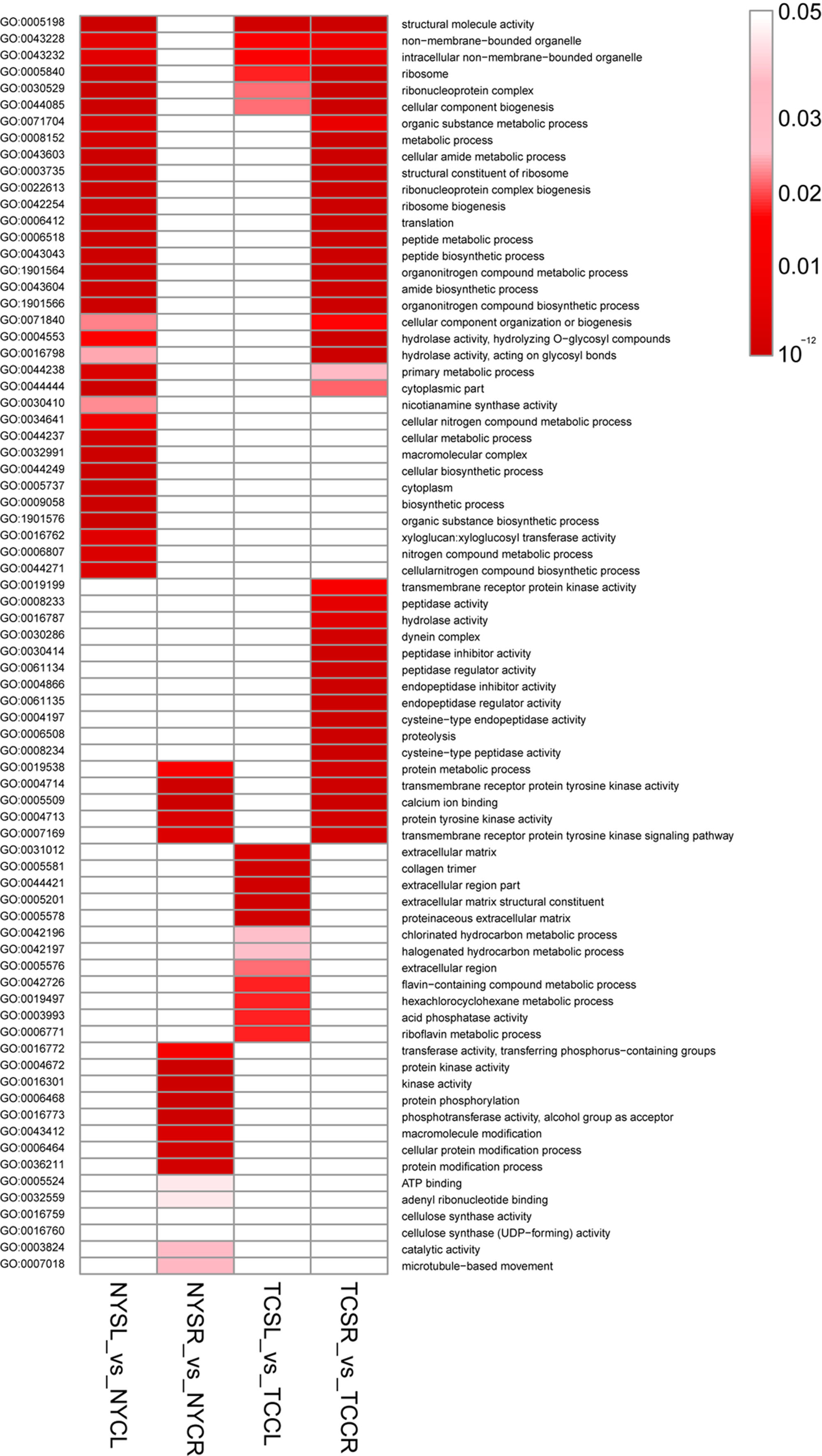

Supplement: Supplementary file 1 [file genes-08-00226-s001.zip › Supplementary Material/Supplementary figure S4.jpg]
